# Rituximab therapy for pure red cell aplasia due to anti-epoetin antibodies in a woman treated with epoetin-alfa: a case report

**DOI:** 10.4076/1752-1947-3-7335

**Published:** 2009-07-06

**Authors:** Caroline M Behler, Norah A Terrault, Joan E Etzell, Lloyd E Damon

**Affiliations:** 1 Department of Hematology and Oncology, University of California, San Francisco, CA, USA; 2 Department of Hematology and Oncology, San Francisco Veterans Affairs Medical Center, San Francisco, CA, USA; 3 Department of Gastroenterology, University of California, San Francisco, CA, USA; 4 Department of Laboratory Medicine, University of California, San Francisco, CA, USA

## Abstract

**Introduction:**

Pure red cell aplasia due to anti-epoetin antibodies is a known complication of epoetin therapy for anemia due to chronic kidney disease. This disease has not previously been well described in the setting of therapy for chronic hepatitis C virus infection. While treatment for pure red cell aplasia due to anti-epoetin antibodies is usually with immunosuppressive therapy such as calcineurin inhibition, the safety of this treatment in chronic hepatitis C virus infection is unknown. To date, little has been published on the efficacy of rituximab on pure red cell aplasia due to anti-epoetin antibodies.

**Case presentation:**

This report describes a 65-year-old Asian-American woman who developed pure red cell aplasia from high titer neutralizing anti-epoetin antibodies after epoetin-alfa therapy during ribavirin and peg-interferon treatment for chronic hepatitis C virus infection. We describe the outcome of her treatment with rituximab. The reticulocyte count increased, and anti-epoetin antibody titer decreased with a loss of neutralizing activity *in vitro*, leading to a reduction in blood transfusions, and eventual resolution of anemia, without reactivation of hepatitis C virus.

**Conclusion:**

The diagnosis of pure red cell aplasia from anti-epoetin antibodies should be considered in patients undergoing therapy for chronic hepatitis C virus infection who develop severe anemia after administration of erythropoietin or darbepoetin. Though it is currently an off-label indication, rituximab is a therapeutic option for patients with pure red cell aplasia due to anti-epoetin antibodies.

## Introduction

Since the introduction of recombinant erythropoietin (r-epoetin) in 1988, an epidemic of pure red cell aplasia (PRCA) due to anti-epoetin antibodies has been identified, with a peak incidence in 2002. The vast majority of patients had anemia from chronic kidney disease (CKD) and were treated in Europe, the UK and Canada [1]. Associations were identified between the development of PRCA and subcutaneous versus intravenous administration, the brand of epoetin, substitution of albumin with polysorbate 80 and glycine as the vehicle [2] and the use of pre-filled syringes with uncoated versus rubber coated plungers [3,4].

The major diagnostic criteria for PRCA due to anti-epoetin antibodies include treatment with epoetin for at least 3 weeks, a red blood cell transfusion requirement of approximately 1 unit per week to keep hemoglobin levels stable, reticulocyte count less than 10 × 10^9^/L and no major drop in white blood cell or platelet counts. Bone marrow biopsy documentation of PRCA and serum assay for neutralizing anti-erythropoietin antibodies are recommended for confirmation of the diagnosis [5].

Treatment of chronic hepatitis C virus (HCV) infection with ribavirin and interferon commonly results in adverse hematologic effects, including hemolytic anemia (ribavirin) and pancytopenia from bone marrow suppression (alfa-interferon). The use of r-epoetin for anemia in this setting has been associated with an increase in hemoglobin levels, higher doses of ribavirin administered and improvement in quality-of-life scores in patients being treated for HCV [6]-[8]. While r-epoetin is commonly used to treat anemia associated with HCV therapy, only one case report of a patient with HCV and r-epoetin-associated PRCA has been published to date [9].

## Case presentation

A 65-year-old Asian-American woman was diagnosed with chronic hepatitis C (HCV), genotype 2 and was referred for treatment of her HCV infection. Abdominal imaging showed a nodular appearance of the liver and hypersplenism with mild thrombocytopenia and leukopenia: baseline white blood cell count 3.2-3.5 × 10^9^/L, neutrophil count 1.2-1.5 × 10^9^/L, hemoglobin 13.5-14.0 g/dL and platelet count 90-100 × 10^9^/L. She was treated with peg-interferon and ribavirin, but therapy was stopped after 3 weeks because of neutropenia and flu-like symptoms. Peg-interferon and ribavirin were re-initiated 8 months later with growth factor support: granulocyte-colony stimulating factor (G-CSF, Neupogen®) 300 μg subcutaneously once to twice weekly and epoetin-alfa (Procrit®) 40,000 units subcutaneously every 1 to 2 weeks. HCV viral load was undetectable by week 4 of therapy. At week 14, she developed severe pancytopenia, with a nadir absolute neutrophil count of 0.4 × 10^9^/L, hemoglobin 6.5 g/dL and platelet count of 20 × 10^9^/L despite continued use of growth factors. Packed red blood cell (PRBC) and platelet transfusions were initiated, and ribavirin was discontinued. Peg-interferon was continued at a reduced dose through week 27, at which time it was stopped. The patient continued to require intermittent transfusions between weeks 14 and 27 of treatment despite continued therapy with epoetin-alfa 40,000 units subcutaneously twice a week. HCV RNA was undetectable at the end of treatment.

Two months after discontinuation of anti-HCV therapy, the patient was referred to Hematology for persistent anemia. At that time, she denied any bleeding or jaundice, and her main symptoms were fatigue and dyspnea on exertion. On physical examination, she had normal vital signs, no jaundice, ecchymoses or petechiae, no lymphadenopathy and palpable hepatosplenomegaly. Laboratory examinations revealed: white blood cell count 4.0 × 10^9^/L, neutrophil count 1.6 × 10^9^/L, hemoglobin 8.9 g/dL, mean corpuscular volume 88 fL, platelet count 63 × 10^9^/L, reticulocyte count 2.6 × 10^9^/L (normal range, 26-110 × 10^9^/L) and an erythropoietin level of 34 mIU/mL (normal range 4.1-19.5). A peripheral blood smear showed a reduction in all three hematopoietic cell lines but with normal cellular morphology. Her ferritin level was 2981 μg/L and thyroid stimulating hormone (TSH), vitamin B12 and folate levels were all normal. Lactate dehydrogenase (LDH) was mildly elevated at 189 IU/L (normal range 91-185) and the direct Coomb's test was negative.

**Figure 1:**
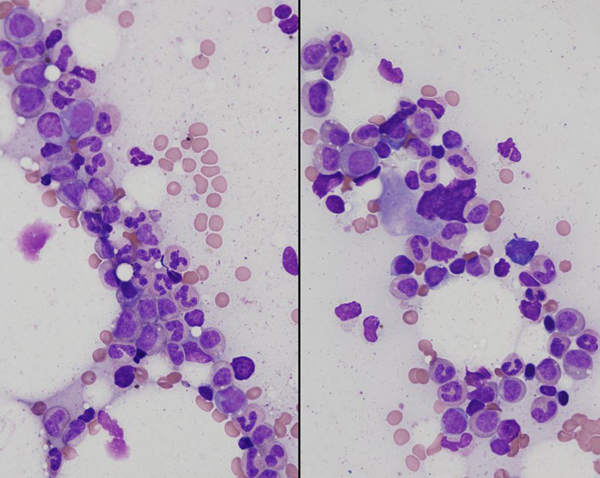
**Bone marrow aspirate**. Bone marrow aspirate showing hypocellularity (overall 10%), near absence of erythroid precursors with full maturation of the myeloid series and normal megakaryocytes.

**Figure 2:**
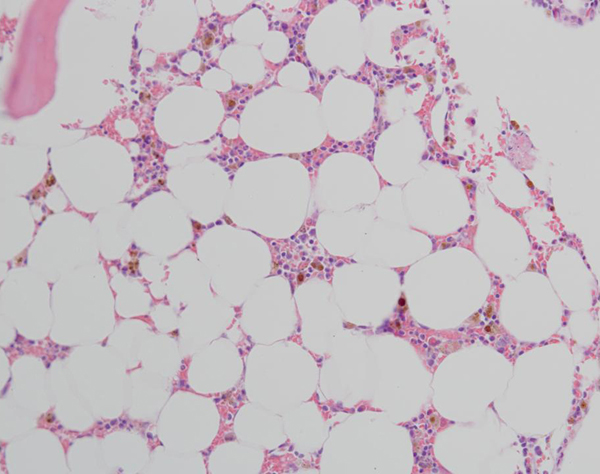
**Bone marrow biopsy**. Core biopsy showing hypocellularity (overall 10%), near absence of erythroid precursors with full maturation of the myeloid series and normal megakaryocytes.

Bone marrow biopsy showed 10% cellularity with profound erythroid hypoplasia, normal myeloid maturation and normal megakaryocytes (Figures 1 and 2). The hypocellularity was thought to be due to prior interferon therapy or possibly HCV infection (though the hepatitis C viral load was undetectable), and the thrombocytopenia was felt to be due to hepatic cirrhosis and hypersplenism. Given the clinical picture of severe anemia and near absent erythroid precursors in the bone marrow, the patient was initially thought to have ribavirin-induced PRCA, and she received two doses of intravenous gammaglobulin (IVIG) 1 g/kg monthly for 2 months, without any hematologic improvement. Analysis of the patient's serum showed IgG1 antibodies to epoetin alfa at a titer of 14.05 μg/mL, which neutralized the biologic activity of epoetin *in vitro*. This confirmed a diagnosis of PRCA due to anti-epoetin antibodies. Epoetin-alfa was discontinued.

**Figure 3:**
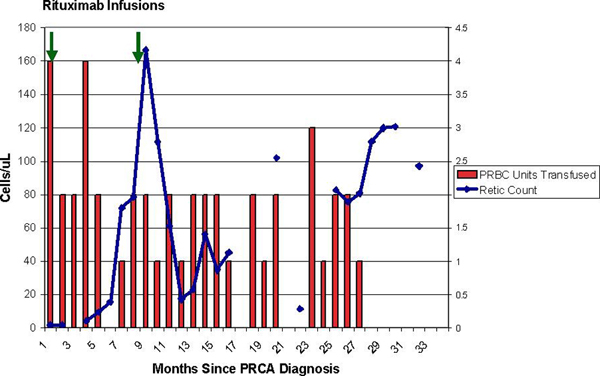
**Reticulocyte and red cell transfusions during rituximab therapy**. Pattern of reticulocyte count and red cell transfusions over time since the diagnosis of pure red cell aplasia. Rituximab 375 mg/m^2^ was given weekly × 4 weeks during months 1 and 9 after diagnosis, as indicated by the vertical arrows at the top of the figure.

The patient then received rituximab 375 mg/m^2^ intravenously weekly for four doses with monthly IVIG 0.5 g/kg for 4 months starting at the time of rituximab therapy. Within 5 months, the reticulocyte count began to increase, which corresponded to a decreasing trend in PRBC transfusions (Figure 3). Eight months after treatment, she again had a drop in her reticulocyte count. The anti-epoetin alfa antibody titer was 7.52 μg/mL and was no longer neutralizing. She was retreated with rituximab at the same dose and schedule as before and there appeared to be another more transient reticulocyte count response, and though this did not translate to a significant change in PRBC transfusions initially, the anemia eventually resolved. Her last blood transfusion occurred 27 months after her initial rituximab course, and her hemoglobin remains stable and normal, 14 months after her last transfusion. The anti-epoetin alfa antibody titer at 37 months after her initial rituximab course was 3.19 μg/mL. The HCV viral load continues to be undetectable and her liver disease remains compensated.

## Discussion

This case demonstrates the potential risk of PRCA due to anti-epoetin antibodies from r-epoetin therapy in patients treated for HCV. Rituximab is a humanized, murine monoclonal antibody that targets the CD20 antigen, leading to antibody-dependent, complement-dependent, cell-mediated cytotoxicity and apoptosis [10]. Case reports and case series describe the successful use of rituximab for the treatment of lymphoproliferative disease-associated PRCA, in association with response in the underlying lymphoproliferative disease [11]. Rituximab has also been reported to induce responses in primary acquired PRCA [12] and in r-epoetin-induced PRCA in a patient with CKD [13].

Because the risk of HCV reactivation with standard immunosuppressive therapy is uncertain, and because of concern for the heightened risk of infectious complications due to the patient's cirrhosis, this patient was treated with rituximab rather than alkylating agents, calcineurin inhibitors or corticosteroids. IVIG was administered because of the possibility of increased risk of HCV reactivation due to B-cell depletion and hypogammaglobulinemia from rituximab therapy; however, there is no published evidence to guide this management.

Most reports of patients with r-epoetin-associated PRCA consist of CKD patients treated outside of the USA. These reports show that spontaneous recovery is rare, but several immunosuppressive treatments can lead to full hematologic recovery and elimination of serum anti-epoetin antibodies. There is little published on the efficacy of rituximab for this disease. A retrospective study examining the outcomes of 47 patients with CKD and r-epoetin-associated PRCA showed no recovery in the 10 patients who stopped epoetin but did not undergo any other therapy [14]. In contrast, 78% of the 37 who received immunosuppressive therapy regained transfusion independence. In 33 patients who had anti-epoetin antibody levels assayed after treatment, recovery only occurred in those who achieved undetectable antibody levels. All six patients who underwent kidney transplantation had resolution of PRCA. In those treated with immunosuppressive therapy alone, the highest response rates were in patients treated with cyclophosphamide plus corticosteroids (7 out of 8 recovered), cyclosporine (4 out of 6), corticosteroids with or without IVIG (10 out of 18) and corticosteroids plus IVIG and plasma exchange (1 out of 1). Recovery from PRCA occurred in only one of nine patients treated with IVIG alone, and in neither of the patients treated with a non-rituximab anti-CD20 monoclonal antibody (0 out of 1) or mycophenylate mofetil (0 out of 1) [14].

A larger series looked at the outcomes of 170 patients diagnosed with r-epoetin-associated PRCA between 1988 and 2004 [15]. Of the 89 patients who were treated with immunosuppressive therapy but did not undergo renal transplant, 49% achieved hematologic recovery (≤1 PRBC transfusions a month, hemoglobin ≥8g/dL and reticulocyte count >20 × 10^9^/L), but only one became transfusion-independent. Of the 33 patients rechallenged with r-epoetin, 56% (and 89% of those with undetectable antibodies) regained r-epoetin-responsiveness. Of the nine patients with neutralizing assay results at the time of rechallenge, all eight without neutralizing antibodies regained r-epoetin-responsiveness, but not the one patient with a positive result [15]. This suggests that even those with detectable antibodies may respond to r-epoetin administration, especially if the antibody is non-neutralizing. However, our patient remained anemic despite the continued presence of an anti-epoetin antibody that does not neutralize epoetin activity *in vitro*, possibly suggesting continued neutralization of endogenous erythropoietin. While this series did not report any successes with anti-CD20 therapy, a single case report of a patient with CKD and r-epoetin-associated PRCA describes erythropoietic recovery following rituximab and rechallenge with r-epoetin [13]. A patient with HCV and r-epoetin-associated PRCA achieved hematologic recovery in 6 weeks after starting danazol [9].

## Conclusion

This case highlights the importance of considering PRCA from anti-epoetin antibodies in patients treated for HCV who develop severe anemia refractory to r-epoetin or darbepoetin administration. This is a well established, but uncommon, consequence of using r-epoetin for the anemia of CKD. Some authors have raised the question as to whether the immune modulatory effects of alpha-interferon therapy might increase the risk of developing anti-epoetin antibodies [9], however this remains speculative. Because the use of recombinant hematopoietic growth factors can ameliorate the hematologic toxicity associated with HCV therapy [6,7], r-epoetin therapy should not be withheld from patients being treated for hepatitis C because of the low, but real, risk of developing PRCA.

Our patient demonstrated initially improved but incomplete erythropoiesis in response to rituximab, then resolution of her anemia more than 2 years after initial treatment, without HCV reactivation. It is not known if she would have eventually achieved spontaneous recovery without treatment, or whether initial depletion of the anti-epoetin-antibody-producing B-cell clone contributed to its eventual extinction.

Rituximab may be considered a therapeutic option in patients with PRCA due to anti-epoetin antibodies; however, the use of rituximab in this setting is outside the scope of its FDA indications. When given to patients at risk for hepatitis B or hepatitis C viral reactivation, caution should be exercised due to the risk of viral reactivation in lymphoma patients receiving rituximab.

## Abbreviations

CKD: chronic kidney disease; G-CSF: granulocyte-colony stimulating factor; HCV: hepatitis C virus; IVIG: intravenous gammaglobulin; LDH: lactate dehydrogenase; PRBC: packed red blood cell; PRCA: pure red cell aplasia; TSH: thyroid stimulating hormone.

## Consent

Written informed consent was obtained from the patient for publication of this case report and any accompanying images. A copy of the written consent is available for review by the Editor-in-Chief of this journal.

## Competing interests

The authors declare that they have no competing interests.

## Authors' contributions

CB, LD and NT were involved in the direct clinical care of the patient. CB and LD analyzed and interpreted the patient's laboratory data and response to therapy. CB wrote the manuscript and LD and NT contributed to manuscript revision. JE performed histological examination of the bone marrow and provided images. All authors read and approved the final manuscript.
